# Specific endophenotypes in EEG microstates for methamphetamine use disorder

**DOI:** 10.3389/fpsyt.2024.1513793

**Published:** 2025-02-03

**Authors:** Xurong Gao, Yun-Hsuan Chen, Ziyi Zeng, Wenyao Zheng, Chengpeng Chai, Hemmings Wu, Zhoule Zhu, Jie Yang, Lihua Zhong, Hua Shen, Mohamad Sawan

**Affiliations:** ^1^ CenBRAIN Neurotech Center of Excellence, School of Engineering, Westlake University, Hangzhou, China; ^2^ Department of Neurosurgery, Second Affiliated Hospital, School of Medicine, Zhejiang University, Hangzhou, China; ^3^ Department of Education and Correction, Zhejiang Gongchen Compulsory Isolated Detoxification Center, Hangzhou, China; ^4^ Zhejiang Liangzhu Compulsory Isolated Detoxification Center, Hangzhou, China

**Keywords:** EEG, microstate, methamphetamine addiction, resting states, detection biomarkers, machine learning, classification

## Abstract

**Background:**

Electroencephalogram (EEG) microstates, which reflect large-scale resting-state networks of the brain, have been proposed as potential endophenotypes for methamphetamine use disorder (MUD). However, current endophenotypes lack refinement at the frequency band level, limiting their precision in identifying key frequency bands associated with MUD.

**Methods:**

In this study, we investigated EEG microstate dynamics across various frequency bands and different tasks, utilizing machine learning to classify MUD and healthy controls.

**Results:**

During the resting state, the highest classification accuracy for detecting MUD was 85.5%, achieved using microstate parameters in the alpha band. Among these, the coverage of microstate class A contributed the most, suggesting it as the most promising endophenotype for specifying MUD.

**Discussion:**

We accurately categorize the endophenotype of MUD into different sub-frequency bands, thereby providing reliable biomarkers.

## 1 Introduction

Methamphetamine use disorder (MUD) can lead to significant impairments in brain function (1). During methamphetamine consumption, dopamine-related neurons are activated, resulting in elevated dopamine levels. This surge produces an exaggerated learning signal that contributes to addiction (2, 3). To address MUD, several medical institutions have proposed therapies such as transcranial magnetic stimulation (TMS) and transcranial direct current stimulation (TDCS). Identifying endophenotypes of MUD is crucial for evaluating the efficacy of these treatments (4). Electroencephalography (EEG), a safe and cost-effective method for recording brain activity in real time, facilitates the study and diagnosis of MUD. Among various analytical methods applied to EEG data, microstate analysis has revealed notable differences in resting-state EEG dynamics between individuals with MUD and healthy controls (HCs, 5, 6).

Microstates are derived from the application of clustering algorithms to EEG data, allowing for segmentation and grouping of similar brain activity patterns. Researchers have discovered that resting-state EEG in the alpha band (8–12 Hz) can be segmented into several quasi-stable states that persist for approximately 60–120 milliseconds before transitioning to another state (7). These quasi-stable phases are thought to reflect the combined transient activity of neuronal clusters in the brain and are often referred to as “the atoms of thought” (8). Over the past two decades, significant associations have been identified between microstate parameters and various neuropsychiatric disorders (9). For instance, a reduced duration of microstates has been noted in studies on depression (10). Additionally, various alterations in microstate dynamics have been reported in Alzheimer’s disease (11), schizophrenia (12), frontotemporal dementia (13), and several other conditions.

EEG microstates typically manifest in the form of topographical maps and are commonly categorized into four distinct topographical patterns: microstate A, B, C, and D. In MUD research, several studies have reported abnormalities in the temporal dynamics of EEG microstates in patients with MUD compared to controls, aiming to identify endophenotypes specific to MUD (5, 14, 15). One study indicated significant differences in the majority of microstate parameters between patients with MUD and HCs, highlighting the potential for exploring MUD-specific features (14). Another study found that MUD patients exhibited shorter mean durations of microstate A and B, as well as a higher global explained variance for microstate C (5). Although these studies, which utilized mixed-band (2–20 Hz) EEG data, have identified several endophenotypes characterizing MUD, mixed-band EEG analysis lacks the precision to distinguish variations in each sub-frequency band affected by different physiological states. Furthermore, the importance of specific microstate parameters was not adequately addressed in previous research, leaving key parameters that reflect MUD unidentified.

Therefore, in this study, we investigated the discriminative power of EEG microstates for MUD across different sub-frequency bands using machine learning techniques. Due to the inherent sensitivity of machine learning to input features, we were able to characterize and rank the importance of microstate parameters, identifying those with the greatest potential to serve as specific endophenotypes of MUD. Additionally, unlike other studies that rely solely on resting-state tasks, we also incorporated visual induction tasks to broaden the scope for identifying MUD endophenotypes.

This paper is organized as follows: Section 2 concerns the adopted materials and methods, Section 3 includes the results, we provide a discussion in Section 4, and conclusions are the subject of Section 5.

## 2 Methods

### 2.1 Participants

**Figure 1:**
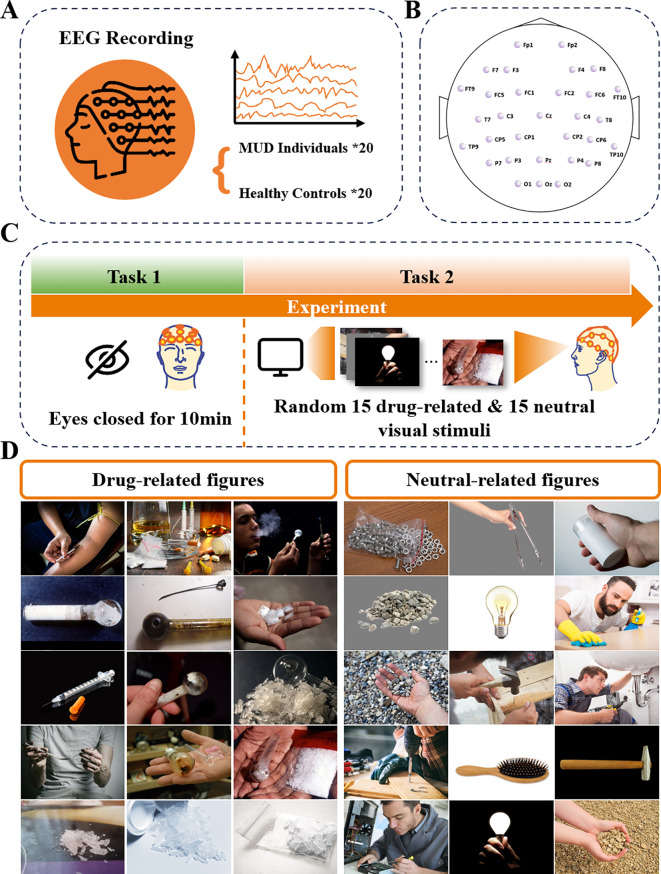
Illustration of experiment protocol: **(A)** MUD and HC group were included, with 20 participants in each group. **(B)** 32-channel EEG data was collected. **(C)** Schematic experimental process: The experiment began with Task 1, a 10-minute resting state task with eyes closed. Task 2 showed 15 drug-related and 15 neutral visual stimuli to the participants. **(D)** The drug-related and neutral figures used in this experiment. Images reprinted with permission from R. Kuplicki, https://github.com/rkuplicki/LIBR_MOCD.

In this study, we recruited 20 male patients with MUD from Gongchen Rehabilitation Center in Zhejiang, China (**Figure 1A**). The recruitment criteria were as follows: (1) exclusive methamphetamine use (single-drug users only), (2) met the criteria for MUD as outlined in the Diagnostic and Statistical Manual of Mental Disorders, Fifth Edition, (3) normal intelligence with no history of head injury, and (4) right-handedness, (5) no personal or family history of other psychiatric illnesses. Additionally, 20 male HCs were recruited from a university campus through advertisements and word-of-mouth between April and July 2023. The selection criteria for HCs included: (1) no history of drug use, (2) normal intelligence with no history of head injury, and (3) right-handedness, (4) no personal or family history of psychiatric illnesses. All participants provided informed consent and received compensation for their participation.

### 2.2 Protocol

Both groups of participants followed the same experimental protocol, as shown in **Figure 1**. EEG data were collected using 33 Ag/AgCl electrodes, including one ground electrode and one reference electrode, arranged according to the 10–20 international standard (**Figure 1B**). The ground electrode was positioned at Fpz, while the reference electrode was placed at Cz. The recording setup consisted of an EEG signal amplifier (actiCHamp Plus, Brain Products) and active EEG electrodes (actiCAP slim, Brain Products), with a sampling frequency of 1000 Hz. An experiment began when the impedance of all channels was below 20 kΩ.

The experimental protocol (**Figure 1C**) was designed using E-Prime software, and EEG signals were recorded under two different tasks for both groups. In Task 1, participants were asked to sit in a plastic chair with a backrest, lean comfortably against it, keep their eyes closed, and remain awake for 10 minutes, minimizing body movements. The EEG signals recorded during this task are defined as resting-state EEG. In Task 2, participants were exposed to 30 pictures, 15 neutral and 15 drug-related, presented randomly. Each picture was considered a separate trial for analysis. The pictures (**Figure 1D**) were selected from the Methamphetamine and Opioid Cue Database (MOCD), with the drug-related images validated to significantly induce craving in participants with MUD (16). During visual stimulation, each image was displayed for 7 seconds, followed by a 7-second black screen before the next image appeared. The random presentation order prevented participants from predicting the type of stimulus, maintaining the element of surprise. Additionally, participants were instructed to press a key before each image appeared to initiate the next trial, ensuring active engagement throughout the experiment. This protocol was approved by the ethical committee of Westlake University (ID: 20191023swan001).

### 2.3 EEG signals processing

For EEG data preprocessing, the raw data were bandpass filtered between 4–45 Hz using a finite impulse response filter. The EEG signals were then down-sampled to 250 Hz to reduce computational load for subsequent independent component analysis (ICA). Bad channels, identified as spectral outliers, were interpolated using an average method. ICA was then applied to remove artifacts such as eye blinks and muscle movements. No bad epochs were found requiring additional manual adjustment. The EEG signals were segmented into epochs based on individual participants, tasks, and four sub-frequency bands: theta (4–7 Hz), alpha (8–12 Hz), beta (13–28 Hz), and gamma (29–45 Hz). For Task 1, 15 epochs, each lasting 7 seconds, were extracted from the first 105 seconds in 10-minute resting-state EEG. For Task 2, each epoch also lasted 7 seconds, corresponding to the duration when the pictures were displayed.

A total of 1,800 all-band (filtered at 4~45Hz) EEG epochs were obtained from the 40 participants, spanning three conditions (resting state, drug-related cue, neutral cue) and 15 trials per condition. After that, another 7200 epochs of separated EEG band were extracted from these 1800 epochs by dividing them into four frequency bands for further analysis. The extraction of different EEG frequency bands was achieved using a finite impulse response filter. All data preprocessing was performed using MATLAB R2013a (The MathWorks, Inc., Natick, USA) with the EEGLAB 2021.0 toolbox.

### 2.4 Microstate analysis

**Figure 2:**
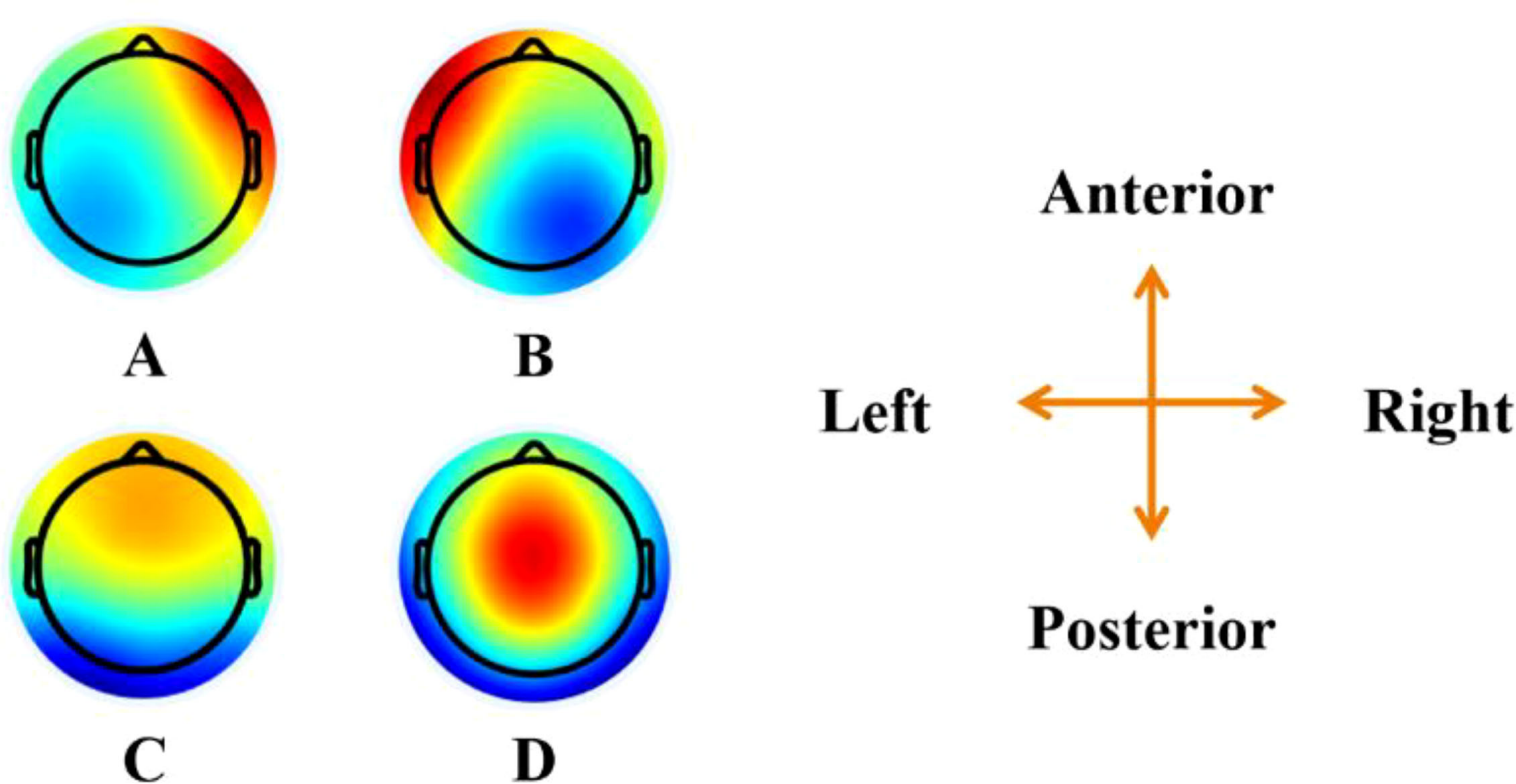
Topographical microstate topographies for both groups. The figure shows the spatial configuration of the four microstate classes **(A–D)**. The top view maps of each class are presented.

Microstate analysis included several steps: global field power calculation, microstate clustering, selection of the number of microstates, back-fitting, labeling, and parameter calculation (17). These processes were conducted using the MATLAB toolbox +microstate (18). We selected and merged the all-band (4~45Hz) EEG data from both groups of participants to conduct microstate analysis, generating the common microstate topographies as shown in **Figure 2**. These topographies were then backfitted to each individual’s EEG data (7,200 epochs for 4 bands and 1,800 epochs for all bands) to obtain their respective microstate parameters. In addition to the all-band EEG microstate topographies, band-specific (theta, alpha, beta, gamma) EEG microstate topographies were also generated, revealing similar patterns to the all-band results (**Supplementary Figure S1**).

After determining the microstate topographies, parameters such as coverage, duration, occurrence, and global explained variance (GEV) for each microstate class were derived. Coverage represents the percentage of time occupied by each microstate, duration indicates the average time span of each microstate, occurrence refers to the number of times each microstate class occurs per second, and GEV reflects the accuracy in explaining the overall sequence of microstate topographies (14).

Previous studies performed microstate clustering separately for each group of participants, but no significant differences were found in the microstate topographies derived from each group (6, 14, 19). Therefore, to compare parameters under consistent microstate topographic conditions and to reduce the potential for invalid features due to differences in microstates, we combined the EEG data from both groups for clustering to obtain common microstate topographies. In this study, four microstate classes, as shown in **Figure 2**, were clustered and selected for further analysis. These patterns closely resemble those reported in related studies, enabling comparative analysis across research. Seventeen parameters derived from the microstates were characterized in this study: the coverage, duration, occurrence, and GEV of the four microstate classes, as well as the total GEV of all microstate classes. In total, microstate parameters were generated from 9,000 EEG epochs, each epoch lasts 7 seconds. These 9,000 epochs included data from five frequency bands (theta, alpha, beta, gamma, and all-band), three task conditions (resting state, drug cue, and neutral cue), 15 trials per condition, and 40 participants. These parameters were used as the dataset for classification using artificial intelligence algorithms.

### 2.5 Random forest classification

To investigate the diagnostic efficacy of MUD under different tasks and identify potential biomarkers of MUD within microstates, we employed a classification approach to analyze microstate data. Given that our aim was not to optimize the algorithm itself, we used Scikit-learn, a widely adopted machine learning library, to perform a random forest classification (20). In this model, the features were the 17 parameters derived from microstate analysis, while the two participant groups served as labels. A five-fold cross-validation was implemented to evaluate the stability of predictions and the generalization ability of the model.

To ensure the validity of the classification, outliers in the microstate parameters were identified and excluded. Specifically, epochs with a duration of zero or exceeding 250 ms were removed, as the duration of a single microstate typically averages between 60 and 100 ms (9). Additionally, due to the varying scales of different parameters, it was essential to normalize each parameter individually to ensure equal weighting among features.

### 2.6 Out-of-Bag analysis

Out-of-Bag (OOB) analysis is a technique used to assess the generalization performance of ensemble learning algorithms, particularly random forests. The key idea is to use the samples not selected for training a specific tree (OOB samples) to evaluate that tree’s performance. In random forests, approximately one-third of the data is excluded from each bootstrap sample, and these OOB samples are leveraged to assess model performance and feature importance. Following classification, an out-of-bag analysis was conducted to rank the importance of each feature.

## 3 Results

### 3.1 Demographic and clinical information of participants

The detailed demographic and clinical information for both groups are presented in **Table 1**.

**Table 1:** Group average statistics of MUD patients and HCs.

Group	MUD patients	HCs	P-value
Age (years)	36.90(7.72)	26.10(4.2)	< 0.01
Age of first use	35.70(8.20)	–	
Quitting times	1.65(0.99)	–	
Gender(male/female)	20/0	20/0	

Group average statistics (Standard Deviation) of MUD patients, HCs. “Age of first use” represents the age at first drug use, and “Quitting times” indicates the number of times forced to quit drugs in rehabilitation centers.

Given the distinct characteristics of the recruitment locations, the average ages of the two groups differed. However, Koenig et al. (21) reported no significant differences in EEG microstate parameters between age groups of 20 to 30 years and 30 to 40 years, suggesting that age-related effects on microstate analysis are negligible in this context.

To rigorously verify that age does not influence microstate parameters, we conducted ANCOVA tests on both groups, while age was included as a covariate. Microstate parameter values from all 15 trials per participant were averaged to be analyzed. In the ANCOVA analysis, the covariate Age did not exhibit a significant main effect on microstate parameters (p > 0.05). Furthermore, the interaction effect between Group and Age was also non-significant (p > 0.05), indicating that the influence of Age on microstate parameters was consistent across the two groups. Results from the MUD group in the Alpha band under resting state are presented in **Table 2** as an example.

**Table 2:** One-way ANCOVA results for the age effect in microstate parameters in alpha band.

Parameter	Age				Age × Group			
	sum_sq	df	F	PR(>F)	sum_sq	df	F	PR(>F)
A_Duration	109.1893	1	0.1011	0.7542	7.4938	1	0.0069	0.9345
B_Duration	543.0562	1	0.6304	0.4376	1028.0870	1	1.1934	0.2891
C_Duration	8.0561	1	0.0114	0.9161	345.2767	1	0.4896	0.4931
D_Duration	2096.9021	1	0.9480	0.3431	446.9988	1	0.2021	0.6584
A_Coverage	0.0060	1	0.7389	0.4013	0.0000	1	0.0000	0.9985
B_Coverage	0.0001	1	0.0131	0.9102	0.0043	1	0.4977	0.4896
C_Coverage	0.0028	1	0.5325	0.4750	0.0065	1	1.2230	0.2833
D_Coverage	0.0144	1	1.0337	0.3228	0.0002	1	0.0148	0.9046
A_Occurrence	0.1235	1	1.2671	0.2751	0.0182	1	0.1866	0.6709
B_Occurrence	0.0027	1	0.0170	0.8977	0.0211	1	0.1309	0.7217
C_Occurrence	0.0958	1	1.0247	0.3248	0.1620	1	1.7325	0.2046
D_Occurrence	0.1348	1	0.7034	0.4126	0.0139	1	0.0725	0.7908
A_GEV	0.0059	1	0.4974	0.4897	0.0026	1	0.2230	0.6425
B_GEV	0.0070	1	0.9347	0.3464	0.0008	1	0.1099	0.7440
C_GEV	0.0022	1	0.8012	0.3826	0.0022	1	0.8204	0.3770
D_GEV	0.0060	1	1.4255	0.2480	0.0000	1	0.0085	0.9274
GEV	0.0080	1	2.4832	0.1325	0.0022	1	0.6730	0.4227

All the significant results have P-values greater than 0.05, suggesting neither Age nor its interaction with Group substantially contributed to the variability in microstate parameters. PR(>F) is the p-value that tests the significance of the F-statistic.

### 3.2 Microstate analysis results

Typically, the duration and GEV of microstates are used to evaluate the fitting performance of microstate analysis, which is highly significant. Our results for duration and GEV, as shown in **Table 3**, indicate that most durations are around 100 ms, and most GEV values range between 0.65 and 0.8, both falling within the normal range. Additionally, our microstate topography, frequency band selection, and GEV results are consistent with those reported in most studies focusing on non-polarized microstates (22, 23). This indicates that our microstate analysis is reliable. Each parameter was calculated as the mean and standard deviation across 15 trials, with all data available in the **Supplementary Tables**.

**Table 3:** Part of microstate parameters results.

Band/Condition		A_Duration	B_Duration	C_Duration	D_Duration	GEV
Theta	MUD-D	130.11 (± 41.97)	101.42 (± 31.68)	91.62 (± 29.87)	83.82 (± 24.08)	0.78 (± 0.06)
	MUD-N	133.36 (± 42.47)	99.44 (± 27.45)	87.64 (± 25.65)	84.48 (± 26.48)	0.78 (± 0.04)
	MUD-R	130.84 (± 34.57)	117.03 (± 33.23)	90.24 (± 20.81)	87.77 (± 22.5)	0.77 (± 0.05)
	HC-D	119.09 (± 31.61)	112.2 (± 28.13)	83.59 (± 21.01)	93.64 (± 27.58)	0.75 (± 0.03)
	HC-N	121.14 (± 32.57)	108.23 (± 24.44)	87.09 (± 22.9)	82.65 (± 24.35)	0.75 (± 0.03)
	HC-R	121.68 (± 31.18)	116.33 (± 23.54)	85.82 (± 18.98)	92.55 (± 20.01)	0.75 (± 0.03)
Alpha	MUD-D	158.64 (± 40.38)	137.95 (± 41.22)	128.12 (± 41.31)	136.08 (± 44.06)	0.73 (± 0.05)
	MUD-N	164.67 (± 38.28)	137.12 (± 38.35)	129.0 (± 37.76)	127.44 (± 44.1)	0.73 (± 0.04)
	MUD-R	182.2 (± 36.87)	160.38 (± 40.35)	140.94 (± 37.47)	136.59 (± 43.47)	0.73 (± 0.05)
	HC-D	159.55 (± 38.31)	144.06 (± 34.32)	115.08 (± 34.14)	132.52 (± 43.42)	0.72 (± 0.04)
	HC-N	171.46 (± 39.1)	139.78 (± 34.0)	123.21 (± 33.08)	119.79 (± 38.81)	0.72 (± 0.04)
	HC-R	183.84 (± 38.29)	162.03 (± 34.7)	118.65 (± 30.23)	157.77 (± 40.21)	0.72 (± 0.03)
Beta	MUD-D	106.35 (± 31.4)	79.14 (± 19.58)	79.83 (± 18.9)	76.74 (± 16.79)	0.69 (± 0.05)
	MUD-N	111.2 (± 34.87)	76.71 (± 16.08)	78.05 (± 18.68)	76.81 (± 17.88)	0.69 (± 0.04)
	MUD-R	120.1 (± 42.9)	83.29 (± 26.25)	77.99 (± 18.57)	77.23 (± 13.42)	0.71 (± 0.06)
	HC-D	101.98 (± 25.0)	84.24 (± 16.73)	74.25 (± 15.4)	83.98 (± 22.25)	0.67 (± 0.04)
	HC-N	111.29 (± 30.13)	80.95 (± 15.42)	76.58 (± 15.19)	78.84 (± 18.88)	0.67 (± 0.04)
	HC-R	120.98 (± 36.43)	83.66 (± 17.19)	74.95 (± 13.79)	89.91 (± 24.3)	0.68 (± 0.05)
Gamma	MUD-D	107.47 (± 35.83)	74.15 (± 22.91)	77.5 (± 18.28)	72.25 (± 14.95)	0.67 (± 0.06)
	MUD-N	108.96 (± 35.97)	69.86 (± 13.85)	78.93 (± 20.56)	74.17 (± 18.01)	0.67 (± 0.05)
	MUD-R	112.93 (± 40.8)	77.95 (± 23.25)	76.07 (± 17.22)	71.32 (± 10.65)	0.68 (± 0.06)
	HC-D	102.78 (± 24.05)	77.65 (± 16.37)	71.98 (± 12.46)	76.63 (± 14.22)	0.64 (± 0.04)
	HC-N	105.73 (± 26.89)	76.14 (± 16.32)	73.95 (± 13.49)	71.82 (± 15.49)	0.64 (± 0.04)
	HC-R	100.55 (± 22.81)	80.86 (± 17.12)	71.29 (± 9.58)	75.83 (± 13.31)	0.65 (± 0.04)
All	MUD-D	111.01 (± 38.25)	76.55 (± 19.33)	76.75 (± 18.38)	74.86 (± 18.93)	0.66 (± 0.05)
	MUD-N	109.64 (± 28.65)	83.89 (± 17.21)	74.61 (± 17.15)	72.38 (± 15.56)	0.65 (± 0.04)
	MUD-R	130.56 (± 45.8)	94.57 (± 26.67)	79.98 (± 20.41)	78.04 (± 21.4)	0.69 (± 0.06)
	HC-D	111.11 (± 31.59)	82.84 (± 16.33)	74.44 (± 14.28)	71.91 (± 17.72)	0.65 (± 0.04)
	HC-N	109.65 (± 37.29)	76.96 (± 17.1)	74.94 (± 16.83)	74.72 (± 19.88)	0.66 (± 0.05)
	HC-R	143.5 (± 42.99)	96.48 (± 27.25)	78.3 (± 15.9)	84.08 (± 21.72)	0.69 (± 0.05)

In this table, the frequency range of each band is Theta (4~8Hz), Alpha (8~12Hz), Beta (12~28Hz), Gamma (28~45Hz), and All (4~45Hz). MUD represents the MUD patient group while the HC denotes the healthy control group. D, N, R means different tasks in this work, they are Drug Cue (D), Neutral Cue (N), Resting State (R). A, B, C, D represents four different microstates. The unit of Duration is milliseconds, while GEV is unitless.

### 3.3 Classification of different tasks for MUD and HCs group

**Figure 3:**
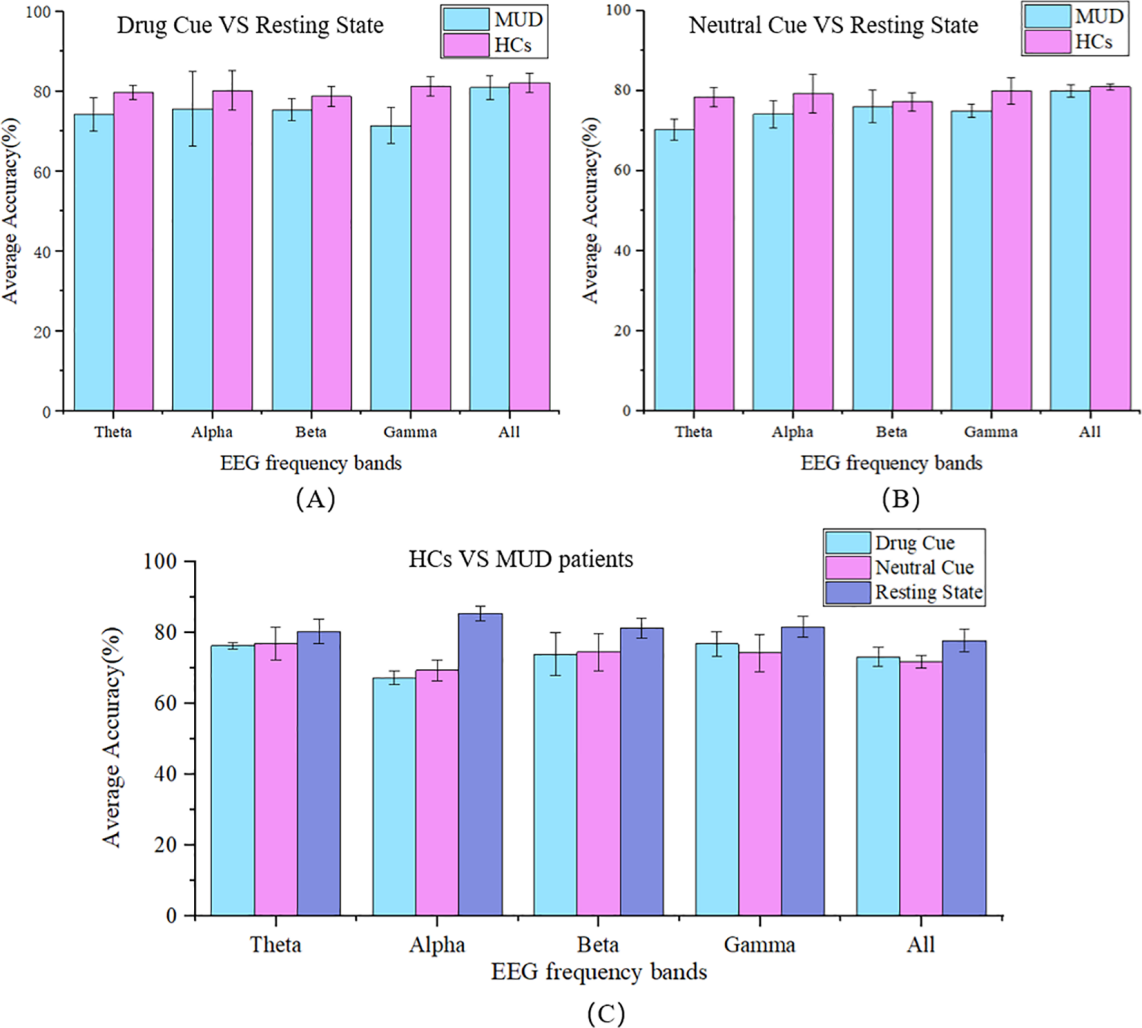
The 5-fold classification accuracy. Classification between resting and visual-stimulation states, separated into **(A)** during the drug-related; and **(B)** neutral cues, for both the MUD and HC groups across different EEG frequency bands (Theta, Alpha, Beta, Gamma, and All). **(C)** Classification between HCs and MUD patients under 3 conditions (Drug cue, Neutral cue, Resting State) across different EEG frequency bands. MUD data represent patients with MUD, HCs data represent healthy controls. The frequency range of each band is Theta (4~8Hz), Alpha (8~12Hz), Beta (12~28Hz), Gamma (28~45Hz), and All (4~45Hz).

To explore the differences in neural and cognitive functions between individuals with MUD and HCs, we used a random forest classifier to analyze resting-state and task-state EEG data, which were further categorized into drug stimuli and neutral stimuli for both groups separately, as shown in **Figures 3A, B**.

### 3.4 Classification of MUD patients and HCs

In the previous section, we observed differences in classification accuracy between MUD patients and HCs. Building on this, we aimed to identify specific biomarkers that could distinguish these two groups. To pinpoint the most representative biomarkers for MUD, the classification was further subdivided by each frequency band and condition (drug cue, neutral cue, and resting state), as **Figure 3C**.

In this binary classification, the average 5-fold cross-validation accuracy for distinguishing between MUD patients and HCs ranged from 67.79% to 85.50%, indicating significant differences in microstate features. In lower frequency bands, such as the theta band, the differences in classification accuracy between different conditions were minimal, at 3.05%. However, in the alpha band, the accuracy difference was substantial, reaching 17.71%. In the beta and gamma bands, this difference gradually decreased to 9.02% and 7.42%, respectively. In the full frequency range (4~45 Hz), the difference was 7.43%. Notably, the highest classification accuracy, 85.5% (± 1.84%), was observed in the alpha band during the resting state, while the lowest accuracy, 67.79% (± 1.86%), occurred in the alpha band during drug stimuli. These findings suggest that the alpha band is the most critical frequency band for distinguishing between MUD patients and HCs.

### 3.5 Feature importance of microstate parameters

Given that the highest accuracy was achieved in the alpha band during the resting state, we conducted an OOB analysis to evaluate the importance of each feature in this classification. This analysis aimed to identify the critical parameters that contribute most to distinguishing between MUD patients and HCs. The feature importance rankings are presented in **Table 4**. The importance of features was not expressed in specific units because the numerical values were directly tied to the magnitude of the data in the dataset. However, these values effectively reflected the comparative relationships between features. In the feature importance ranking, the coverage of microstate A ranked highest with an importance score of 0.1190, followed closely by the global explained variance (GEV) of microstate A at 0.1054. These features were the most critical contributors to achieving the highest classification accuracy of 85.5% for distinguishing between MUD patients and HCs during the resting state.

**Table 4:** Feature importance of microstate parameters.

No	Microstate	Parameter	Importance	No	Microstate	Parameter	Importance
1	A	Coverage	0.1190	10	C	GEV	0.0478
2	A	GEV	0.1054	11	D	Duration	0.0425
3	B	GEV	0.1028	12	B	Duration	0.0422
4	A	Occurrence	0.0805	13	C	Coverage	0.0403
5	Total	GEV	0.0765	14	B	Occurrence	0.0336
6	A	Duration	0.0641	15	B	Coverage	0.0322
7	D	Coverage	0.0624	16	C	Duration	0.0228
8	D	Occurrence	0.0578	17	C	Occurrence	0.0160
9	D	GEV	0.0533	–	–	–	–

Microstate topography of A, B, C, and D are presented in **Figure 2**. Parameters of microstate are introduced in Section 2.4 (Microstate Analysis). Total-GEV means the total global explained variance of four microstates. Importance represents the importance of each feature.

## 4 Discussions

### 4.1 Cognitive difference between MUD Patients and HCs

In our classification results regarding different tasks (Section 3.1), we found that the classification accuracy for distinguishing between the visual stimuli task (drug stimuli or neutral stimuli) and the resting state was higher in the HC group compared to the MUD group across all frequency bands. These results suggested significant differences in the dynamics of neuronal networks and indicated cognitive differences between MUD patients and HCs. This phenomenon maybe attributed to long-term methamphetamine abuse, which can lead to neural damage and disrupt the brain’s functional connectivity (24). Such disruptions alter the brain’s response patterns to external stimuli and contribute to cognitive functional impairment (25). Previously, structural and functional differences between drug addicts and healthy individuals in brain imaging (5, 26–28) particularly in functional magnetic resonance imaging (fMRI, 29), were readily identified through microstate analysis based on these structural disparities (30). In our study, all these structural and functional differences in the brain, leading to cognitive differences, were reflected in the data features of microstate analysis and were visually demonstrated through machine learning classification.

### 4.2 Key EEG band for specific endophenotypes for MUD

The main purpose of this study was to explore the specific endophenotypes of MUD. The first objective was to precisely identify the most MUD-related EEG frequency band, where we aimed to explore the key parameters of microstates as specific endophenotypes for MUD. In a previous review (31), it was noted that the majority of current research on microstates predominantly selects EEG frequency bands ranging from 2 to 20 Hz or 2 to 40 Hz. There is currently no fully unified standard or consensus regarding the selection of EEG frequency bands for microstate analysis. Similarly, in studies focusing on addiction-related microstate analysis, most EEG microstate research integrates different frequency bands into a unified analysis, encompassing multiple subdivided frequency ranges (32, 33). This approach reflects the general dynamics of neuronal networks but does not help to identify specific bands with key neuronal activity. In contrast, neuroscience research, including studies on MUD, often targets specific EEG frequency bands to investigate their unique physiological roles (34). For example, a power increase was found in the beta and gamma bands in patients with MUD compared to HCs (35). Consequently, in this work, we studied not only aggregated bands but also each frequency band (theta, alpha, beta, gamma) individually. Our results revealed that segmented EEG frequency bands for MUD detection yielded much higher average accuracies (with 85.50% as the highest among all sub-frequency bands) than detecting MUD by aggregating all frequency bands together (79.11%) under resting state conditions. The highest accuracy was observed in the alpha band (8-12 Hz). In other words, the performance for classifying patients with MUD and HCs reached its maximum when considering only the alpha frequency band. Compared to the delta, beta, and gamma frequency bands, the average accuracy improved by 3.66%, suggesting that the alpha frequency band has the highest priority. Although other frequency bands also exhibited high classification accuracy (82.97% in the beta band and 82.29% in the gamma band under resting state), indicating that they contain crucial information for distinguishing between the two groups, their stability, as indicated by the standard deviation performance, was on average 0.98% lower.

The current understanding of the EEG alpha band has not been fully elucidated (36); however, it is generally believed that the alpha band is strongly associated with memory (37), creativity (38), and cognitive abilities (39). In microstate analysis, the importance of the alpha band in exploring MUD endophenotypes is now being discussed for the first time. However, the potential of the alpha band as a characterization for MUD has already been demonstrated in some previous studies. Most MUD-related EEG research has reported the specificity of the alpha band (35, 40–43). For example, under eye-closed resting state conditions, patients with MUD exhibit lower alpha band power in the EEG spectrum compared to HCs (40, 43). Another example is the increase in alpha band power observed in MUD patients when transitioning from a drug-related to a neutral VR task (35). These studies align closely with our characterization of the internal endophenotype of MUD, to some extent demonstrating the importance of the alpha band. However, unlike previous research where the alpha band was treated as a biomarker, in this work, it serves as the primary band for further exploration of the MUD endophenotype. Furthermore, we focused on the alpha band to explore the most crucial microstate parameters as specific endophenotypes of MUD in the resting state.

### 4.3 Key parameters as specific endophenotypes for MUD

In our feature importance rank, microstate A emerged as the primary contributor, followed by microstate D, while the contributions of microstates C and B were considerably weaker. This indicates that microstate A has the strongest association with MUD (**Table 4**). Microstate A most effectively reflects the differences between MUD patients and HCs. It is noteworthy that these four microstate topographies are highly reproducible, with most studies adopting similar microstates, facilitating comparisons across research.

Each microstate class has specific physiological significance. For example, microstate class A is generally associated with speech and auditory processing (44). Additionally, microstate A has been linked to schizophrenia (45), depression (46), various types of addiction (33, 47) and a high risk of developing mental disorders (48). Our previous work (49) reviewed microstate analysis in MUD and found that patients with MUD tend to exhibit relatively lower coverage in microstate class A (14). This finding is consistent with the importance ranking of microstate A coverage in our out-of-bag analysis, where it ranked highest (**Table 4**). Moreover, similar neurodynamic abnormalities have been observed in studies of heroin addiction (6), suggesting that this phenotype may have broader relevance in representing various forms of drug addiction.

## 5 Comparison across different studies and limitations

A comparison of various studies focusing on drug addiction-related microstate analysis is presented in **Table 5**. Most research using EEG microstate analysis in addiction studies has identified specific biomarkers (5, 14) and some have proposed the use of repetitive transcranial magnetic stimulation to treat MUD patients (6, 15). One study, however, focused on improving addiction detection accuracy through novel algorithms without identifying specific biomarkers (50). Despite these advancements, the critical frequency sub-bands characterizing MUD have not been adequately emphasized or identified in the existing literature. Our study addresses this gap by exploring the role of specific frequency bands in MUD. Additionally, the investigation of EEG microstates under different task conditions is relatively rare (14). Unlike typical resting-state studies, our research introduces visual induction tasks involving drug-related and neutral images to examine their effects on microstate analysis for MUD. Furthermore, our study achieved an addiction detection accuracy rate of 85.5% for methamphetamine addiction, representing notable improvement over previous research (6, 26).

**Table 5:** A Comparison of studies that investigate drug-craving with microstate approach.

Authors	Drugs	EEG freq. bands	Sub-bands analysis	Different tasks	Therapies	Biomarkers	MUD vs HCs Classification	Accuracy
Chen (5)	Meth	2-20	×	×	×	√	×	–
Ding (6)	Heroin	2-20	×	×	√	√	√	81.5%
Lin (14)	Meth	2-20	×	√	×	√	×	–
Li (15)	Meth	0.1-45	×	×	√	√	×	–
Wang (50)	Heroin	2-20	×	×	×	×	√	81.0%
This work	Meth	4-45	√	√	×	√	√	85.5%

Different tasks mean resting state and visual stimuli. Therapies refer to rTMS. Biomarkers, namely endophenotypes for MUD detection. "√" indicates the item is accomplished, while "×" indicates the item is not included.

This work highlights several areas for potential improvement. Firstly, enhancing the experimental stimulation by incorporating virtual reality (VR) and drug-like objects could elicit more distinct EEG patterns, thereby providing a clearer understanding of addiction mechanisms. Secondly, some experimental designs and analytical methods could be improved. It would be more rigorous if the sample size were further expanded, female participants were included, and the age differences between groups were balanced. Meanwhile, the random 5-fold cross-validation approach may allow trials from the same subject to appear in both the training and validation sets, introducing feature leakage and exacerbating confounding effects. This setup may cause the model to learn subject-specific characteristics rather than true differences between MUD and HC, leading to an overestimated accuracy. A subject-wise 5-fold split would better mitigate this issue. Thirdly, to validate the proposed biomarkers, therapeutic interventions could be explored to treat MUD patients and assess significant changes in these biomarkers. Implementing closed-loop neuromodulation may also help validate our proposed biomarkers and improve therapeutic outcomes for MUD patients. At last, while our machine learning approach identified key microstate parameters (coverage and GEV in the alpha band) as strongly associated with MUD, this method primarily reveals patterns based on data rather than directly addressing the disease’s intrinsic characteristics. Thus, there remains a gap in interpretability, and further research is needed to explore the physiological significance of these identified endophenotypes.

## 6 Conclusions

This study integrates microstate analysis with machine learning to identify the EEG frequency band most relevant to MUD. The alpha band emerged as strongly associated with MUD, with microstate class A proving to be the most effective in distinguishing MUD patients from HCs. Additionally, the duration of microstate class A was found to be the most critical parameter for classification, suggesting its potential as a key endophenotype for MUD.

## Data Availability

The data that support the findings of this study are available from the corresponding author, Y. Chen, upon reasonable request.
