# Supplementary material for: Specific endophenotypes in EEG microstates for methamphetamine use disorder
Source: Front Psychiatry. 2025 Feb 3;15:1513793. doi: 10.3389/fpsyt.2024.1513793 (PMC11831278; doi:10.3389/fpsyt.2024.1513793)
Supplement: Supplementary file 1 [file Supplementaryfile1.docx]

Supplementary Material

Supplementary Table I. Microstate Parameters of the MUD group under Drug Cue

| Drug Cue | MUD | | | | |
| --- | --- | --- | --- | --- | --- |
| Parameters | Theta | Alpha | Beta | Gamma | All |
| A_Duration | 130.11 (±41.97) | 158.64 (±40.38) | 106.35 (±31.4) | 107.47 (±35.83) | 111.01 (±38.25) |
| B_Duration | 101.42 (±31.68) | 137.95 (±41.22) | 79.14 (±19.58) | 74.15 (±22.91) | 76.55 (±19.33) |
| C_Duration | 91.62 (±29.87) | 128.12 (±41.31) | 79.83 (±18.9) | 77.5 (±18.28) | 76.75 (±18.38) |
| D_Duration | 83.82 (±24.08) | 136.08 (±44.06) | 76.74 (±16.79) | 72.25 (±14.95) | 74.86 (±18.93) |
| A_Coverage | 0.37 (±0.15) | 0.33 (±0.12) | 0.38 (±0.14) | 0.4 (±0.16) | 0.4 (±0.16) |
| B_Coverage | 0.25 (±0.11) | 0.26 (±0.12) | 0.21 (±0.11) | 0.19 (±0.11) | 0.21 (±0.1) |
| C_Coverage | 0.2 (±0.12) | 0.2 (±0.12) | 0.21 (±0.11) | 0.23 (±0.12) | 0.2 (±0.11) |
| D_Coverage | 0.18 (±0.11) | 0.22 (±0.11) | 0.19 (±0.09) | 0.19 (±0.09) | 0.19 (±0.12) |
| A_Occurrence | 2.81 (±0.75) | 2.04 (±0.5) | 3.51 (±0.8) | 3.59 (±0.87) | 3.53 (±0.8) |
| B_Occurrence | 2.46 (±0.67) | 1.8 (±0.53) | 2.61 (±0.83) | 2.44 (±0.9) | 2.68 (±0.86) |
| C_Occurrence | 2.03 (±0.86) | 1.46 (±0.57) | 2.54 (±0.98) | 2.77 (±1.09) | 2.52 (±0.96) |
| D_Occurrence | 2.03 (±0.95) | 1.53 (±0.56) | 2.42 (±0.91) | 2.52 (±0.99) | 2.34 (±1.06) |
| A_GEV | 0.29 (±0.16) | 0.23 (±0.12) | 0.25 (±0.13) | 0.25 (±0.14) | 0.25 (±0.13) |
| B_GEV | 0.14 (±0.08) | 0.14 (±0.09) | 0.1 (±0.06) | 0.08 (±0.06) | 0.09 (±0.06) |
| C_GEV | 0.11 (±0.1) | 0.09 (±0.08) | 0.09 (±0.06) | 0.1 (±0.07) | 0.08 (±0.07) |
| D_GEV | 0.09 (±0.08) | 0.1 (±0.07) | 0.08 (±0.05) | 0.07 (±0.05) | 0.08 (±0.06) |
| GEV' | 0.78 (±0.06) | 0.73 (±0.05) | 0.69 (±0.05) | 0.67 (±0.06) | 0.66 (±0.05) |

Supplementary Table II. Microstate Parameters of the MUD group under Neutral Cue

| Neutral Cue | MUD | | | | |
| --- | --- | --- | --- | --- | --- |
| Parameters | Theta | Alpha | Beta | Gamma | All |
| A_Duration | 133.36 (±42.47) | 164.67 (±38.28) | 111.2 (±34.87) | 108.96 (±35.97) | 109.64 (±28.65) |
| B_Duration | 99.44 (±27.45) | 137.12 (±38.35) | 76.71 (±16.08) | 69.86 (±13.85) | 83.89 (±17.21) |
| C_Duration | 87.64 (±25.65) | 129.0 (±37.76) | 78.05 (±18.68) | 78.93 (±20.56) | 74.61 (±17.15) |
| D_Duration | 84.48 (±26.48) | 127.44 (±44.1) | 76.81 (±17.88) | 74.17 (±18.01) | 72.38 (±15.56) |
| A_Coverage | 0.39 (±0.15) | 0.35 (±0.12) | 0.4 (±0.14) | 0.41 (±0.16) | 0.4 (±0.12) |
| B_Coverage | 0.25 (±0.09) | 0.25 (±0.11) | 0.2 (±0.09) | 0.17 (±0.08) | 0.25 (±0.09) |
| C_Coverage | 0.2 (±0.11) | 0.2 (±0.11) | 0.21 (±0.11) | 0.23 (±0.12) | 0.19 (±0.08) |
| D_Coverage | 0.16 (±0.12) | 0.2 (±0.12) | 0.19 (±0.11) | 0.2 (±0.11) | 0.17 (±0.1) |
| A_Occurrence | 2.91 (±0.69) | 2.09 (±0.49) | 3.56 (±0.72) | 3.66 (±0.78) | 3.61 (±0.67) |
| B_Occurrence | 2.48 (±0.67) | 1.77 (±0.51) | 2.54 (±0.78) | 2.34 (±0.79) | 2.91 (±0.74) |
| C_Occurrence | 2.22 (±0.85) | 1.47 (±0.52) | 2.52 (±0.94) | 2.77 (±1.03) | 2.45 (±0.78) |
| D_Occurrence | 1.78 (±0.99) | 1.5 (±0.55) | 2.39 (±0.98) | 2.51 (±1.0) | 2.2 (±1.01) |
| A_GEV | 0.3 (±0.15) | 0.25 (±0.12) | 0.26 (±0.13) | 0.25 (±0.14) | 0.25 (±0.11) |
| B_GEV | 0.14 (±0.08) | 0.14 (±0.08) | 0.09 (±0.06) | 0.07 (±0.05) | 0.11 (±0.05) |
| C_GEV | 0.11 (±0.09) | 0.09 (±0.07) | 0.09 (±0.07) | 0.1 (±0.07) | 0.07 (±0.04) |
| D_GEV | 0.08 (±0.09) | 0.1 (±0.07) | 0.08 (±0.06) | 0.08 (±0.06) | 0.06 (±0.06) |
| GEV' | 0.78 (±0.04) | 0.73 (±0.04) | 0.69 (±0.04) | 0.67 (±0.05) | 0.65 (±0.04) |

Supplementary Table III. Microstate Parameters of the MUD group under Resting State

| Resting State | MUD | | | | |
| --- | --- | --- | --- | --- | --- |
| Parameters | Theta | Alpha | Beta | Gamma | All |
| A_Duration | 130.84 (±34.57) | 182.2 (±36.87) | 120.1 (±42.9) | 112.93 (±40.8) | 130.56 (±45.8) |
| B_Duration | 117.03 (±33.23) | 160.38 (±40.35) | 83.29 (±26.25) | 77.95 (±23.25) | 94.57 (±26.67) |
| C_Duration | 90.24 (±20.81) | 140.94 (±37.47) | 77.99 (±18.57) | 76.07 (±17.22) | 79.98 (±20.41) |
| D_Duration | 87.77 (±22.5) | 136.59 (±43.47) | 77.23 (±13.42) | 71.32 (±10.65) | 78.04 (±21.4) |
| A_Coverage | 0.35 (±0.12) | 0.34 (±0.09) | 0.41 (±0.16) | 0.41 (±0.17) | 0.42 (±0.15) |
| B_Coverage | 0.3 (±0.12) | 0.28 (±0.11) | 0.22 (±0.12) | 0.21 (±0.12) | 0.25 (±0.13) |
| C_Coverage | 0.18 (±0.1) | 0.2 (±0.11) | 0.18 (±0.11) | 0.21 (±0.12) | 0.17 (±0.1) |
| D_Coverage | 0.17 (±0.09) | 0.18 (±0.09) | 0.18 (±0.09) | 0.17 (±0.08) | 0.16 (±0.1) |
| A_Occurrence | 2.6 (±0.53) | 1.86 (±0.36) | 3.38 (±0.68) | 3.5 (±0.72) | 3.23 (±0.57) |
| B_Occurrence | 2.52 (±0.52) | 1.69 (±0.43) | 2.56 (±0.81) | 2.59 (±0.86) | 2.58 (±0.79) |
| C_Occurrence | 1.92 (±0.75) | 1.39 (±0.46) | 2.21 (±0.89) | 2.55 (±0.99) | 2.03 (±0.81) |
| D_Occurrence | 1.88 (±0.7) | 1.25 (±0.39) | 2.24 (±0.88) | 2.35 (±0.86) | 1.88 (±0.93) |
| A_GEV | 0.26 (±0.13) | 0.25 (±0.1) | 0.29 (±0.15) | 0.27 (±0.15) | 0.29 (±0.14) |
| B_GEV | 0.19 (±0.11) | 0.16 (±0.1) | 0.11 (±0.07) | 0.1 (±0.08) | 0.13 (±0.1) |
| C_GEV | 0.08 (±0.07) | 0.09 (±0.08) | 0.08 (±0.07) | 0.08 (±0.07) | 0.06 (±0.06) |
| D_GEV | 0.08 (±0.06) | 0.07 (±0.05) | 0.07 (±0.05) | 0.06 (±0.04) | 0.06 (±0.05) |
| GEV' | 0.77 (±0.05) | 0.73 (±0.05) | 0.71 (±0.06) | 0.68 (±0.06) | 0.69 (±0.06) |

Supplementary Table IV. Microstate Parameters of the HC group under Drug Cue

| Drug Cue | HC | | | | |
| --- | --- | --- | --- | --- | --- |
| Parameters | Theta | Alpha | Beta | Gamma | All |
| A_Duration | 119.09 (±31.61) | 159.55 (±38.31) | 101.98 (±25.0) | 102.78 (±24.05) | 111.11 (±31.59) |
| B_Duration | 112.2 (±28.13) | 144.06 (±34.32) | 84.24 (±16.73) | 77.65 (±16.37) | 82.84 (±16.33) |
| C_Duration | 83.59 (±21.01) | 115.08 (±34.14) | 74.25 (±15.4) | 71.98 (±12.46) | 74.44 (±14.28) |
| D_Duration | 93.64 (±27.58) | 132.52 (±43.42) | 83.98 (±22.25) | 76.63 (±14.22) | 71.91 (±17.72) |
| A_Coverage | 0.32 (±0.11) | 0.35 (±0.12) | 0.35 (±0.11) | 0.38 (±0.12) | 0.4 (±0.13) |
| B_Coverage | 0.3 (±0.1) | 0.28 (±0.09) | 0.25 (±0.09) | 0.23 (±0.1) | 0.24 (±0.09) |
| C_Coverage | 0.16 (±0.08) | 0.15 (±0.07) | 0.17 (±0.07) | 0.18 (±0.08) | 0.19 (±0.08) |
| D_Coverage | 0.22 (±0.11) | 0.22 (±0.12) | 0.23 (±0.12) | 0.22 (±0.09) | 0.17 (±0.1) |
| A_Occurrence | 2.67 (±0.59) | 2.16 (±0.49) | 3.42 (±0.69) | 3.65 (±0.7) | 3.63 (±0.63) |
| B_Occurrence | 2.68 (±0.61) | 1.95 (±0.4) | 2.92 (±0.74) | 2.82 (±0.86) | 2.9 (±0.78) |
| C_Occurrence | 1.88 (±0.72) | 1.31 (±0.45) | 2.19 (±0.77) | 2.4 (±0.83) | 2.44 (±0.72) |
| D_Occurrence | 2.32 (±0.81) | 1.57 (±0.56) | 2.65 (±0.93) | 2.75 (±0.86) | 2.18 (±1.01) |
| A_GEV | 0.23 (±0.11) | 0.24 (±0.11) | 0.22 (±0.1) | 0.22 (±0.09) | 0.25 (±0.11) |
| B_GEV | 0.17 (±0.08) | 0.15 (±0.08) | 0.12 (±0.05) | 0.1 (±0.05) | 0.11 (±0.05) |
| C_GEV | 0.07 (±0.05) | 0.06 (±0.04) | 0.06 (±0.04) | 0.06 (±0.04) | 0.07 (±0.04) |
| D_GEV | 0.12 (±0.09) | 0.11 (±0.08) | 0.1 (±0.07) | 0.08 (±0.05) | 0.06 (±0.06) |
| GEV' | 0.75 (±0.03) | 0.72 (±0.04) | 0.67 (±0.04) | 0.64 (±0.04) | 0.65 (±0.04) |

Supplementary Table V. Microstate Parameters of the HC group under Neutral Cue

| Neutral Cue | HC | | | | |
| --- | --- | --- | --- | --- | --- |
| Parameters | Theta | Alpha | Beta | Gamma | All |
| A_Duration | 121.14 (±32.57) | 171.46 (±39.1) | 111.29 (±30.13) | 105.73 (±26.89) | 109.65 (±37.29) |
| B_Duration | 108.23 (±24.44) | 139.78 (±34.0) | 80.95 (±15.42) | 76.14 (±16.32) | 76.96 (±17.1) |
| C_Duration | 87.09 (±22.9) | 123.21 (±33.08) | 76.58 (±15.19) | 73.95 (±13.49) | 74.94 (±16.83) |
| D_Duration | 82.65 (±24.35) | 119.79 (±38.81) | 78.84 (±18.88) | 71.82 (±15.49) | 74.72 (±19.88) |
| A_Coverage | 0.35 (±0.12) | 0.37 (±0.11) | 0.4 (±0.12) | 0.41 (±0.13) | 0.4 (±0.16) |
| B_Coverage | 0.29 (±0.1) | 0.26 (±0.09) | 0.22 (±0.08) | 0.22 (±0.09) | 0.21 (±0.09) |
| C_Coverage | 0.2 (±0.09) | 0.18 (±0.08) | 0.19 (±0.08) | 0.2 (±0.08) | 0.2 (±0.1) |
| D_Coverage | 0.17 (±0.09) | 0.18 (±0.11) | 0.19 (±0.1) | 0.18 (±0.1) | 0.19 (±0.12) |
| A_Occurrence | 2.85 (±0.59) | 2.18 (±0.41) | 3.55 (±0.66) | 3.8 (±0.7) | 3.61 (±0.81) |
| B_Occurrence | 2.66 (±0.66) | 1.87 (±0.45) | 2.69 (±0.74) | 2.76 (±0.87) | 2.71 (±0.83) |
| C_Occurrence | 2.22 (±0.71) | 1.45 (±0.49) | 2.48 (±0.77) | 2.65 (±0.8) | 2.48 (±0.97) |
| D_Occurrence | 1.95 (±0.78) | 1.43 (±0.58) | 2.31 (±0.92) | 2.35 (±0.98) | 2.37 (±1.04) |
| A_GEV | 0.24 (±0.11) | 0.27 (±0.12) | 0.25 (±0.1) | 0.23 (±0.09) | 0.25 (±0.13) |
| B_GEV | 0.17 (±0.08) | 0.13 (±0.07) | 0.1 (±0.05) | 0.09 (±0.05) | 0.09 (±0.06) |
| C_GEV | 0.09 (±0.06) | 0.07 (±0.05) | 0.07 (±0.04) | 0.08 (±0.04) | 0.08 (±0.06) |
| D_GEV | 0.08 (±0.07) | 0.08 (±0.08) | 0.08 (±0.06) | 0.07 (±0.05) | 0.07 (±0.06) |
| GEV' | 0.75 (±0.03) | 0.72 (±0.04) | 0.67 (±0.04) | 0.64 (±0.04) | 0.66 (±0.05) |

Supplementary Table VI. Microstate Parameters of the HC group under Resting State

| Resting State | HC | | | | |
| --- | --- | --- | --- | --- | --- |
| Parameters | Theta | Alpha | Beta | Gamma | All |
| A_Duration | 121.68 (±31.18) | 183.84 (±38.29) | 120.98 (±36.43) | 100.55 (±22.81) | 143.5 (±42.99) |
| B_Duration | 116.33 (±23.54) | 162.03 (±34.7) | 83.66 (±17.19) | 80.86 (±17.12) | 96.48 (±27.25) |
| C_Duration | 85.82 (±18.98) | 118.65 (±30.23) | 74.95 (±13.79) | 71.29 (±9.58) | 78.3 (±15.9) |
| D_Duration | 92.55 (±20.01) | 157.77 (±40.21) | 89.91 (±24.3) | 75.83 (±13.31) | 84.08 (±21.72) |
| A_Coverage | 0.31 (±0.11) | 0.34 (±0.1) | 0.4 (±0.14) | 0.37 (±0.12) | 0.44 (±0.13) |
| B_Coverage | 0.32 (±0.08) | 0.28 (±0.07) | 0.22 (±0.1) | 0.25 (±0.11) | 0.24 (±0.11) |
| C_Coverage | 0.16 (±0.07) | 0.13 (±0.05) | 0.14 (±0.06) | 0.17 (±0.06) | 0.14 (±0.06) |
| D_Coverage | 0.21 (±0.1) | 0.25 (±0.11) | 0.23 (±0.11) | 0.21 (±0.09) | 0.17 (±0.09) |
| A_Occurrence | 2.52 (±0.49) | 1.84 (±0.4) | 3.29 (±0.59) | 3.59 (±0.54) | 3.1 (±0.54) |
| B_Occurrence | 2.72 (±0.42) | 1.74 (±0.32) | 2.53 (±0.76) | 2.99 (±0.7) | 2.48 (±0.66) |
| C_Occurrence | 1.86 (±0.53) | 1.05 (±0.32) | 1.89 (±0.63) | 2.38 (±0.65) | 1.78 (±0.6) |
| D_Occurrence | 2.16 (±0.68) | 1.53 (±0.46) | 2.5 (±0.76) | 2.67 (±0.76) | 1.94 (±0.71) |
| A_GEV | 0.22 (±0.11) | 0.26 (±0.1) | 0.27 (±0.13) | 0.22 (±0.1) | 0.31 (±0.12) |
| B_GEV | 0.19 (±0.07) | 0.13 (±0.07) | 0.09 (±0.06) | 0.11 (±0.06) | 0.11 (±0.07) |
| C_GEV | 0.07 (±0.04) | 0.04 (±0.03) | 0.05 (±0.03) | 0.06 (±0.03) | 0.04 (±0.03) |
| D_GEV | 0.1 (±0.07) | 0.13 (±0.08) | 0.1 (±0.07) | 0.08 (±0.05) | 0.06 (±0.05) |
| GEV' | 0.75 (±0.03) | 0.72 (±0.03) | 0.68 (±0.05) | 0.65 (±0.04) | 0.69 (±0.05) |

| 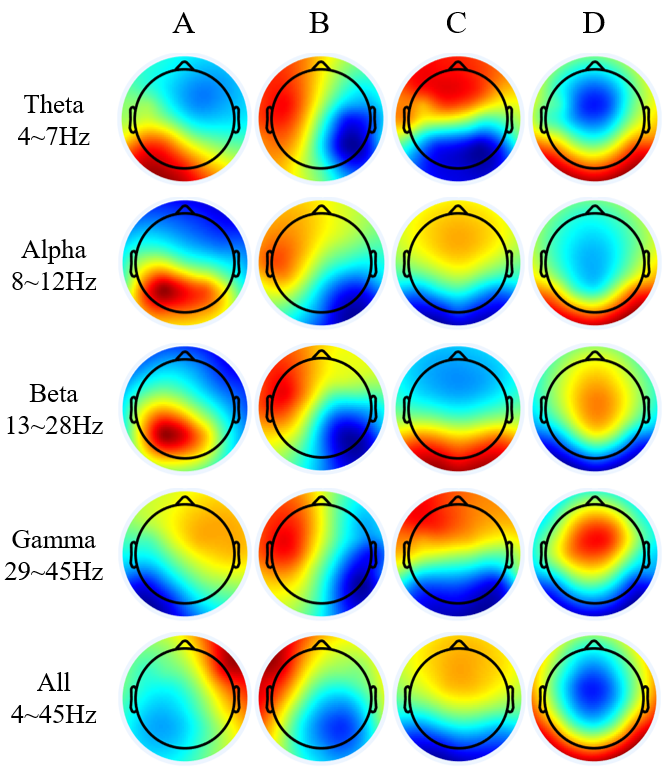 |
| --- |
| **Supplementary Fig. 1** Topographical microstate topographies for both groups in four sub-bands and all bands. The figure shows the spatial configuration of the four microstate classes (A–D). The top view maps of each class are presented. |
